# Social and Medical Determinants of Diabetes: A Time-Constrained Multiple Mediator Analysis

**DOI:** 10.7759/cureus.46227

**Published:** 2023-09-29

**Authors:** Farrokh Alemi, Kyung Hee Lee, Jee Vang, David Lee, Mark Schwartz

**Affiliations:** 1 Health Administration and Policy, George Mason University, Fairfax, USA; 2 Recreation, Parks and Leisure Services Administration, Central Michigan University, Mount Pleasant, USA; 3 Department of Emergency Medicine, New York University Grossman School of Medicine, New York City, USA; 4 Department of Population Health, New York University Grossman School of Medicine, New York City, USA

**Keywords:** diabetes, racial/ethnic disparities, obesity, social determinants of health, time-constrained multiple mediation analysis

## Abstract

Background

A number of studies have shown an association between social determinants of health and the emergence of obesity and diabetes, but whether the relationship is causal is not clear.

Objective

To test whether social, environmental, and medical determinants directly or indirectly affect population-level diabetes prevalence after controlling for mediator-mediator interactions.

Methods

Data were obtained from the CDC and supplemented with nine other data sources for 3,109 US counties. The dependent variable was the prevalence of diabetes in 2017. Independent variables were a given county's 30 social, environmental, and medical characteristics in 2015 and 2016. A network multiple mediation analysis was conducted. First, we used Least Absolute Shrinkage and Selection Operator (LASSO) regression to relate the 2017 diabetes rate in each county to 30 predictors measured in 2016, identifying statistically significant and robust predictors as the mediators within the network model and as direct determinants of 2017 diabetes. Second, each of the direct causes of diabetes was taken as a new response variable and LASSO-regressed on the same 30 independent variables measured in 2015, identifying the indirect (mediated) causes of diabetes. Subsequently, these direct and indirect predictors were used to construct a network model. The completed network was then employed to estimate the direct and mediated impact of variables on diabetes.

Results

For 2017 diabetes rates, 63% of the variation was explained by five variables measured in 2016: the percentage of residents who were (1) obese, (2) African American, (3) physically inactive, (4) in poor health condition, and (5) had a history of diabetes. These five direct predictors, measured in 2016, mediated the effect of indirect variables measured in 2015, including the percentage of residents who were (1) Hispanic, (2) physically distressed, (3) smokers, (4) living with children in poverty, (5) experiencing limited access to healthy foods, and (6) had low income.

Conclusion

All of the direct predictors of diabetes prevalence, except the percentage of residents who were African American, were medical conditions potentially influenced by lifestyles. Counties characterized by higher levels of obesity, inactivity, and poor health conditions exhibited increased diabetes rates in the following year. The impact of social determinants of illness, such as low income, children in poverty, and limited access to healthy foods, had an indirect effect on the health of residents and, consequently, increased the prevalence of diabetes.

## Introduction

Social determinants of health (SDOH) and their association with diabetes have been well-documented. Also, many national and international organizations have developed frameworks to better understand SDOH (e.g., Healthy People 2030). These influences are generally conceptualized in frameworks that depict a reciprocal relationship between an individual and their surroundings' socioeconomic and political circumstances, with various mediating factors involved. Many studies have looked for non-medical predictors of diabetes [1-3]; several have highlighted socioeconomic contributors to diabetes [4,5]; others explored associations between access to quality food stores and diabetes [6]; and some have looked at fast food density [7] or access to green spaces and neighborhood walkability [8]. However, of the 1,170 empirically focused neighborhood effects papers published in the last 24 years, only a handful have clearly advanced our understanding of the phenomena. The independent impact of neighborhood contexts on health remains unclear [9]. The key limitation of previous studies is that they have not actively controlled for medical causes of diabetes. Thus, it is not clear if social determinants of illness have an effect on diabetes independent of medical history. Obesity, physical activity, history of poor health, disability, and smoking behavior are well-known predictors of diabetes. These variables are often omitted as mediating factors in the relationship between social determinants of illness and diabetes prevalence. To reduce overestimation and selection bias, we included these well-known medical factors in our analysis and previously confirmed social determinants of diabetes [10,11]. When both medical and non-medical causes are included in the same analysis, the interaction among these variables becomes central to the analysis. Poverty, for example, can impact lifestyle choices that lead to poor health. Similarly, poor health can lead to less physical activity and weight gain, which lead to obesity and, subsequently, to diabetes. To sort out what is a direct cause of diabetes, what is an indirect cause, and what is not a cause of diabetes, one has to examine the interaction among the independent variables, which is known in the literature as mediator-mediator interactions. Many studies analyze bi-directional relationships; these studies cannot determine how obesity, physical activity, smoking, disability, or social determinants affect diabetes or mediate the effect of each other on diabetes. This study utilizes the temporal order of independent predictors of diabetes to clarify which comes first, medical or social predictors of diabetes. 

## Materials and methods

Study design

This paper uses a network multiple mediation analysis [12]. Traditionally, multiple mediation analysis is done by using coefficients in related regressions; one involves the mediator variables and one that does not [13-16]. The network and the traditional approaches are similar in the sense that both (a) use inverse propensity weights to remove confounding, (b) allow for mediator-mediator interactions, (c) require chronological information about the mediators, and (d) rely extensively on regression analysis [12,17]. We prefer the network modeling approach because it uses the timing of events to resolve mediator-mediator interactions. The knowledge of the timing of events, for example, allows a mediator, such as obesity, to be on the causal pathway from social determinants of illness to diabetes.

Method of analysis

To construct the network model, we repeatedly used the Least Absolute Shrinkage and Selection Operator (LASSO) regressions. The use of LASSO in network modeling is relatively new and was pioneered by Shojaie A and Michailidis G [18,19]. They mathematically proved that when the order of occurrence of variables is known, the likelihood function can be written as an adjacency matrix of the network. Others have also made the same point: LASSO regression selects variables that are on the Markov blanket of the response variable [20-22]. If independent variables in the regressions occur prior to the response variable, then LASSO regressions identify the structure of the network model [18,23]. Due to the wide availability of regression software, regression has been used repeatedly to create directed acyclical networks [12,24-26]. The steps we followed to construct the network model were: 1) We established the order of occurrence of the variables by the year in which the variable was measured; 2) We LASSO-regressed diabetes rates in 2017 on all independent variables measured in 2016. In this regression, the statistically significant and robust variables identify the direct predictors of diabetes. In LASSO, "statistically significant" refers to non-zero coefficients. Robust variables remain significant in 95% of the LASSO regressions on randomly selected data (90% of the whole dataset); and 3) We LASSO-regressed each 2016 direct predictor of diabetes on 2015 independent variables to identify indirect (mediated) predictors of diabetes. 

Once the network structure is identified, regression coefficients can be used to specify the parameters of the network model. The impact of the exposure variable was reported using procedures first described by Pearl J et al. [12]. 

Sources of data 

Our analytical units consist of all 3,109 contiguous US counties. These are currently the smallest analytical units used to incorporate other well-known social/environmental predictors of diabetes across the United States. Analysis was done on the diabetes rate in 3,109 counties over a three-year period from 2015 to 2017. The dependent variable was the 2017 diabetes rate in the county; independent variables were measured in 2015 and 2016. County-level data were obtained from a variety of public/private data sources. Key health variables (e.g., % of diabetic patients, obese population, physically inactive population, mental/physical distress, adult smokers, average mentally/physically healthy days, and general health status) were obtained from the Behavioral Risk Factor Surveillance System coordinated by the CDC. Limited access to a quality food store was based on the percentage of people living more than one mile from a supermarket or large grocery store in an urban area or more than 10 miles in a rural area. The assumption is that supermarkets and large grocery stores provide healthier food options (e.g., fresh fruit and vegetables) than convenience stores or smaller grocery stores [27], especially in low-income areas that are likely to have a higher share of convenience stores and small food markets [28]. The urban-rural classification scheme was obtained from the National Center for Health Statistics. The Natural Amenity Scale was obtained from the United States Department of Agriculture; this scale was constructed by combining six measures of climate, topography, and water that reflect preferred environmental qualities. County proportions of water, forest, and open space were obtained from the United States Geological Survey. County-level spatial information (e.g., county boundaries) was obtained from the Census Bureau's Population Estimates program. Data from the natural amenity index was unavailable for the study years and assumed to be the same as the measure last updated in 1999. Information about quality food access was obtained from the Food Environment Atlas coordinated by the United States Department of Agriculture. Limited access to a quality food store in 2016 was interpolated from 2010 and 2015 data. Food insecurity was obtained from Map the Meal Gap and County Health Rankings; this represents the percentage of residents in the county who did not have access to a reliable source of food in the previous 12 months. The Small Area Income and Poverty Estimates program obtained the percentage of children in poverty. Air quality (>PM2.5), community design, and built environmental data (e.g., park proximity within 0.5 miles) were obtained from the Environmental Public Health Tracking Network coordinated by the CDC. Each county's age, gender, educational attainment, marital status, active commute, poverty, and race/ethnicity distributions were obtained from the American Community Survey.

## Results

In the first step of the analysis, diabetes in 2017 was LASSO-regressed on 30 county characteristics measured in 2016 (see Table 1 for the list of variables). Five variables emerged as non-zero, robust predictors of diabetes in 2017, each with a regression coefficient larger than 0.05, which was the assumed minimum effect size for a clinically meaningful change in the rate of diabetes. These variables were: 1) history of diabetes in the county in 2016; 2) percent of African American residents in the county in 2016; 3) percent of residents with poor health in the county in 2016; 4) percent of obese residents in the county in 2016; and 5) percent of residents physically inactive in the county in 2016. Each of these five variables independently impacted the 2017 rate of diabetes. The remaining 25 variables had no direct or independent impact on diabetes prevalence. Variables without a direct impact included lifestyle variables (e.g., smoking), health history variables (e.g., percent of days with poor physical/mental health), neighborhood variables (e.g., percent of residents with access to quality food), pollution variables (i.e., the daily density of fine particulate matter in the air), and social determinants of illness (e.g., percent of residents unemployed).

**Table 1:** Coefficients in six LASSO regression models. LASSO: Least Absolute Shrinkage and Selection Operator.

Prior Year Independent Variables	Dependent Variables in Six LASSO Regressions					
	% Residents Diabetic in 2017	% African-American Residents in 2016	% Residents Physically Inactive in 2016	% Residents Obese in 2016	% Residents Diabetic in 2016	% Residents Poor or Fair Health in 2016
% Residents African American	0.08	0.97	.	.	.	.
% Residents Hispanic	.	.	.	.	.	0.11
% Residents Diabetic	0.63	.	.	.	0.55	.
% Residents Obese	0.12	.	.	0.74	.	.
% Residents Physically Inactive	0.06	.	0.77	.	.	0.09
% Residents Physical Distressed	.	.	.	.	.	0.17
% Residents in Poor or Fair Health	0.11	.	.	.	.	0.56
% Residents who Smoke	.	.	.	.	.	0.07
% Residents with Children in Poverty	.	.	.	.	.	0.13
% Residents with Limited Access to Healthy Foods	.	.	.	-0.05	.	.
Average Residents’ Income	.	.	-0.07	.	-0.06	.
Notes: The following variables were incorporated as independent variables in the regression analysis but did not yield statistical significance in any of the regressions: % unemployed, % located near a park (within .5 mile), % forested area, % water area, % open space, the daily density of fine particulate matter in the air, presence of high natural amenities, large population, large central metro, large fringe metro, medium metro, small metro, largest town with population between 10,000 and 50,000, largest town with population under 10,000, % food insecure, % uninsured adults, % uninsured children, % of days with poor mental health, % of days with poor physical health, % mentally distressed, % White, % American Indian and Alaskan Native, % Asian, % Native Hawaiian or Pacific Islander, % not proficient in English, % female, % member of associations, % aged 65 or older, and % aged 18 or younger.						

**Figure 1:**
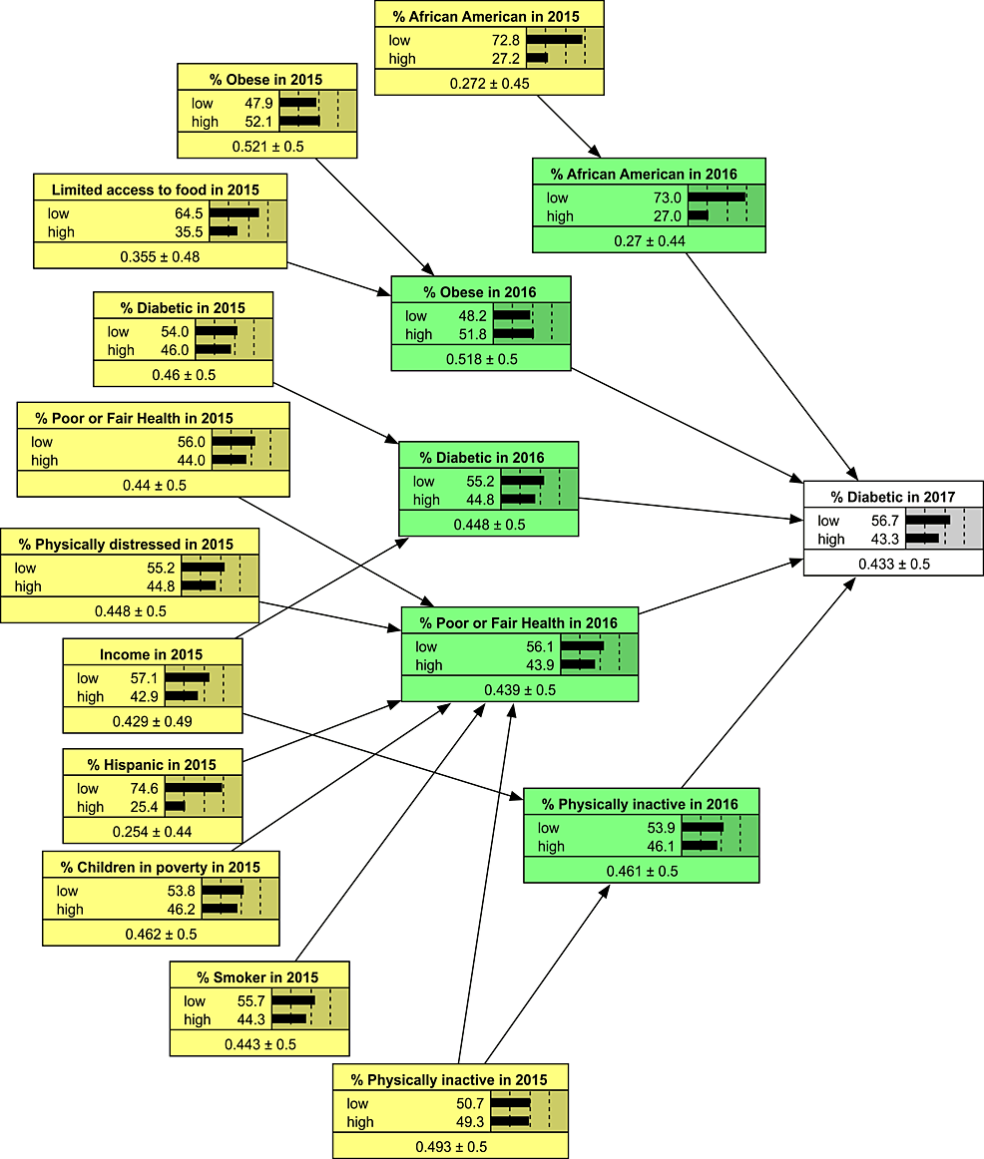
Network model of diabetes over two years. Notes to Figure: The Network Model depicts variables measured at different time points. The outcome of interest, measured in 2017, is displayed to the right. The study evaluated the influence of 30 variables, including % living within 0.5 miles of a park, % physical inactivity, % across different ages, % in poor or fair health, % smokers, % across different races, % participating in social associations, % days of air pollution, % unemployment, % uninsured, % children in poverty, % water area, % with a history of diabetes, % female, median household income, % food insecure, number of days with poor mental health, % forest area, number of days with poor physical health, % Hispanic, total population, limited access to healthy foods, natural amenity scale, % experiencing mental distress, metro size, % not proficient in English, % obese, % open space, and % experiencing physical distress. These variables were assessed in both 2015 and 2016. The network only displays variables with a non-zero (significant) correlation to diabetes. All variables were binary, represented as either above or below the average value of the variable in the county. The binary distribution of each variable is indicated beneath its title, followed by the average proportion and confidence interval.

This initial analysis established the direct 2016 predictors of diabetes in 2017. The five variables identified were also potential meditators for variables measured in 2015. The second step verified the mediation effect through five logistic LASSO regressions. In each regression, one of the five 2016 mediators was selected as the response variable (results also shown in Table 1). The response variable was binarized (set to 1 if above average and 0 otherwise). In all regressions, the independent variables remained the same 30 variables but were now measured in 2015. Once more, statistically significant variables in 9 out of 10 cross-validated LASSO regressions with a coefficient larger than 0.05 were selected, as detailed in Table 1. For instance, the 2016 percentage of African American residents in a county was regressed on 30 variables measured in 2015, with the only statistically significant and robust predictor being the same variable measured in the previous year. This regression suggests that one-year changes in the racial composition of the county are not influenced by the other variables examined. None of the environmental, social, medical, or economic variables, including income level, impacted the racial composition of the county. Similar regressions were conducted for the percentage of physically inactive residents, the percentage of obese residents, the percentage of diabetic residents, and the percentage of residents in poor or fair health. Table 1 shows the results of these regressions, which identify indirect predictors of diabetes. A large number of variables measured in 2015 affected the percentage of residents in poor or fair health in 2016. These included: percentage of Hispanic residents in 2015; percentage of residents physically inactive in 2015; percentage of residents physically distressed in 2015; percentage of residents in poor or fair health in 2015; percentage of residents who smoked in 2015; and percentage of residents with children in poverty in 2015. In the final step, the structure of the network (i.e., the link between variables and the direction of the link) was specified. The absolute values for coefficients in the LASSO regression were used to create links between the variables in the network. Variables not linked to the network were ignored. The direction of the link was set by the year of the measure: earlier measures pointed towards later measures. Figure 1 shows the network structure. This network shows both direct and mediated variables (indirect predictors) of diabetes in 2017. The parameters of this network structure were estimated using Netica software. To estimate the parameters, all continuous variables were broken into binary variables by setting values above average to 1, and values below average to 0. Netica estimated the probability associated with changes in one binary variable impacting another.

## Discussion

The aim of this study was to identify both direct and indirect factors influencing diabetes prevalence. The study is unique in how it actively incorporated the temporal order of independent variables so that the effects of medical and social factors can be separated. This allowed us to effectively eliminate confounders and specify the direct and indirect predictors of diabetes. From the first set of LASSO regressions, the study identified five direct predictors from 2016 data that impacted diabetes rates in 2017: 1) Percent of Black residents; 2) Percent of residents with poor health; 3) Percent of physically inactive residents; 4) Percent of obese residents; and 5) Percent of diabetic patients in 2016. These direct variables were supported by previous literature on diabetes studies [29-33] and explained 63% variation in the diabetes prevalence in 2017. As we expected, medical factors, obesity, physical activity, and general health conditions were highly associated with diabetes. The proportion of African-American residents also had a direct impact on diabetes. One possible explanation for this could be a genetic connection between race and diabetes, independent of social determinants, income, and the other variables studied here. Similar findings have been reported in other studies as well [33]. Contrary to our expectations, 25 other well-known predictors of diabetes­, including economic factors such as income, neighborhood factors such as limited access to quality food stores, and environmental factors such as pollution, were not direct predictors of diabetes prevalence in 2017. The indirect predictors of diabetes were discovered through repeated LASSO regression models, where the direct predictor of diabetes was now the response variable in the regression, and the independent variables were the variables in the prior year. Six new indirect predictors of diabetes have been identified, each supported by the literature. This study, along with others, has found that the quality of food access impacts the prevalence of diabetes [34]. Similarly, it has been established that socioeconomic conditions, particularly income, play a role [35,36]. Additionally, rates of children in poverty [37] have been shown to indirectly affect diabetes prevalence. The percentage of residents who smoke [38] and the percentage of Hispanic residents [39] also indirectly influence diabetes rates. These findings clarify the interactions between the medical and social variables traditionally used to explain diabetes. Many existing studies that analyze large cohorts or county-level data do not consider the temporal order of the independent variables and suggest that all direct/indirect variables consistently correlate with diabetes. This study showed that economic factors do not directly cause diabetes but rather influence diabetes indirectly after affecting the health of the individual, e.g., after physical inactivity or after obesity. These data suggest that social indicators may be the root causes of poor health, and it might be reasonable for policymakers to address these root causes and health issues. This study has investigated the influence of socioeconomic, environmental, and medical determinants on diabetes over a brief interval. We recognize that employing a one-year timeframe to assess direct impacts and two years for indirect ones may be insufficient. There is sparse literature on the duration required for diabetes to manifest due to unhealthy lifestyle habits or specific neighborhood characteristics. It is widely understood that diabetes typically develops over decades, attributable to both direct and indirect risk factors. For instance, adverse childhood experiences such as food insecurity have been demonstrated to result in diabetes three to seven decades later [40,41]. With these decade-long processes, one could argue that the timeframe adopted in the study is too short. At the same time, several social determinants (e.g., average income level in the county or percent of children in poverty) have a statistically significant, indirect effect on diabetes within a short timeframe. Therefore, some social determinants seem to have an impact more quickly, while others may have longer lag times. Future studies should expand the timeframe to further examine the relationship between structural factors of racial/ethnic disparities and diabetes prevalence. The study findings should not be interpreted to suggest that a healthy person will develop diabetes within a year or two of exposure to these factors. These findings simply indicate the annual contribution of social determinants over a long period (exactly how long is unclear). The results indicate the short-term contributions of these factors without refuting the long-term progression of the disease, which is driven by the cumulative effect of the factors over time. This study employed county-level data to illustrate neighborhood effects, acknowledging that within counties exist a multitude of neighborhoods, differing significantly in income and social conditions. Investigating more granular data at the census tract- or individual patient level could yield different insights. Comparative studies among county-level, census tract-level, and individual-level analyses reveal only moderate agreement between these measures [42]. The interpretation would gain robustness by focusing on the smallest analytical unit (i.e., the individual) rather than aggregating results across diverse county populations. Employing this methodological approach proves especially fruitful when integrating electronic health records (EHRs) with neighborhood data [43]. The findings of this study hinge on the validity of the measures used. Errors in measuring the social, economic, environmental, and medical determinants of diabetes may lead to different conclusions. It is also possible that factors not included in our study may predict variations in diabetes prevalence (e.g., political circumstances) [44]. We encourage further studies to consider other social and medical variables not examined in this paper. The fact that our model explained a large percentage of variation in diabetes rates suggests that we have included many of the important predictors of diabetes.

## Conclusions

This study aimed to investigate the direct and indirect factors influencing diabetes prevalence to enhance causal interpretation. We employed a novel approach by considering the temporal order of independent variables, allowing for the separation of medical and social factors' effects and the elimination of confounding variables. The time of events was utilized to statistically control for mediator-mediator interactions. For the first time, the impacts of multiple mediators on diabetes are reported. This is contrary to previous studies that have examined single mediators.
